# PIK3CA mutations are associated with pathologic complete response rate to neoadjuvant pyrotinib and trastuzumab plus chemotherapy for HER2-positive breast cancer

**DOI:** 10.1038/s41416-022-02021-z

**Published:** 2022-11-02

**Authors:** Qiyun Shi, Juncheng Xuhong, Tao Luo, Jia Ge, Feng Liu, Yang Lan, Qingqiu Chen, Peng Tang, Linjun Fan, Li Chen, Yan Liang, Minghao Wang, Ying Hu, Yi Zhang, Xiuwu Bian, Xiaowei Qi, Jun Jiang

**Affiliations:** 1 Department of Breast and Thyroid Surgery, Southwest Hospital, Army Medical University, 400038 Chongqing, China; 2 Shigatse Branch, Xinqiao Hospital, Army Medical University, 857000 Shigatse, China; 3 Institute of Pathology and Southwest Cancer Center, Southwest Hospital, Army Medical University, 400038 Chongqing, China

**Keywords:** Breast cancer, Predictive markers

## Abstract

**Background:**

Neoadjuvant treatment with a dual anti-human epidermal growth factor receptor 2 (HER2) blockade with pyrotinib and trastuzumab has been shown to be effective for HER2-positive breast cancer.

**Methods:**

The genomic characteristics of 425 cancer-related genes from the archived tumour blocks of 50 patients enrolled in a prospective neoadjuvant pyrotinib and trastuzumab plus chemotherapy clinical trial (ChiCTR1900022293) were assessed by next-generation sequencing (NGS). The relationship between tumour biomarkers and the postoperative pathological complete response (pCR) were explored.

**Results:**

Forty-five patients completed neoadjuvant chemotherapy and final surgery, of which 26 (58%) achieved a pCR. Among all driver gene mutations, PIK3CA mutation was screened out for having a significant relationship with the treatment response. The pCR rate of patients with wild-type PIK3CA was significantly higher than patients with mutated PIK3CA (80.8% vs. 26.3%; *P* = 0.00057), and remained significant after a multiple comparison adjustment (*P*_adjusted_ = 0.024). We further evaluated the predictive value with logistic regression model of clinical features, genetic biomarkers or both, an AUC of 0.912 (95% CI: 0.827−0.997) was achieved in the integrated model.

**Conclusions:**

Our data suggest that HER2-positive breast cancers with activating mutations in PIK3CA are less likely to benefit from pyrotinib combined with trastuzumab neoadjuvant therapy.

## Introduction

Human epidermal growth factor receptor 2 (HER2), also known as ErbB2, is a member of the ErbB family of receptor tyrosine kinases [1]. This family forms a complex multilayer network with its intracellular downstream signalling pathways, notably phosphatidylinositol 3-kinase (PI3K)/Akt and mitogen activate protein kinase (MAPK) pathways [2]. The overexpression of ErbB2 is considered to promote oncogenic processes via triggering dimer formation bias of the ErbB family of receptors and aberrantly activating downstream pathways [1].

At present, there are three major classes of anti-HER2 therapeutics applied in clinical practice: (1) monoclonal antibodies; (2) antibody–drug conjugates; and (3) small-molecule tyrosine-kinase inhibitors (TKI). Moreover, monoclonal antibodies targeting the extracellular domain of the ErbB2 receptor, (i.e., trastuzumab) have been widely used and significantly improved the outcome of HER2-positive breast cancer. Reported mechanisms of trastuzumab include but are not limited to the downregulation of surface ErbB2 and ErbB2-mediated signalling pathways, as well as the recruitment of immune effector cells [2]. The addition of trastuzumab to neoadjuvant chemotherapy has been shown to lead to a significantly higher pathological complete response (pCR) compared to chemotherapy alone; however, resistance continues to exist in a considerable number of patients [3]. Some mechanisms have been proposed to explain trastuzumab resistance, including truncated ErbB2 protein, compensatory pathways, and signalling aberrations downstream of ErbB2 [4]. Another therapeutic strategy that competes with ATP in the tyrosine-kinase domain to block ErbB2 signalling was developed, and officially approved TKIs include lapatinib, neratinib, and pyrotinib [5, 6]. The non-overlapping mechanisms of two therapeutics confer a theoretical synergistic interaction to anti-tumour activity, which was validated at the clinical level. A meta-analysis of four large randomised controlled trials that examined the neoadjuvant addition of lapatinib to trastuzumab plus chemotherapy in patients with early HER2-positive breast cancer: NeoALTTO, CALGB 40601, NSABP B41, and CHER-LOB, demonstrated that a dual HER2-blockade with trastuzumab and lapatinib was associated with significantly improved outcomes compared to treatment with trastuzumab alone, and could separately reduce the risk of relapse and death by 38% and 35% [7–11].

Pyrotinib is a pan-ErbB TKI that has recently been shown to be clinically effective for the treatment of HER2-positive breast cancer in metastatic and neoadjuvant settings [12–17]. The mechanism of pyrotinib is different with lapatinib, it has the capacity of ireversibly blocking ErbB1, ErbB2, and ErbB4. Accordingly, a Phase 3 randomised controlled study comparing shown that pyrotinib plus capecitabine significantly improved progression-free survival (PFS) compared with that for lapatinib plus capecitabine for the treatment of HER2-positive metastatic breast cancer. In our previous pilot study of neoadjuvant pyrotinib and trastuzumab combined with chemotherapy, a pCR rate of 73.7% (14/19) was observed in unselected early HER2-positive breast cancer [17]. Similar results were found in another ongoing study involving neoadjuvant pyrotinib and trastuzumab plus docetaxel and carboplatin, with a reported pCR rate in the treatment group of 71.4% (15/21) [14]. Although these results are promising, the current conclusion regarding the safety and efficacy of neoadjuvant pyrotinib remains subject to sample size and follow-up time, which require further validation.

Although dual HER2-targeted neoadjuvant therapy of pyrotinib and trastuzumab primarily illustrated a high pCR rate, some patients did not respond or even developed drug resistance. In this regard, it is necessary to identify tumours that are highly responsive to this therapy and develop more effective criteria for patient preselection. To date, there are several biological characteristics implicated in response to anti-HER2 therapy, including the hormone receptor status [18–20], intrinsic subtype [21], immune signatures [22], tumour-infiltrating lymphocytes (TILs) [23], and alterations in signalling pathways downstream of the ErbB2 family (e.g., PI3K/Akt activation) [24–26]. Considering that HER2 overexpression as determined by immunohistochemistry (IHC) or fluorescence in situ hybridisation (FISH) represents the only criteria for dual-targeted anti-HER2 therapy at a molecular level at the present stage, the exploration of prognostic predictors from the above-mentioned biomarkers is recommended.

In this study, we developed a spectrum of somatic alterations from the preoperative biopsy specimens of 50 HER2-positive breast cancer patients via the next-generation sequencing (NGS) of 425 genes. In particular, we conducted an exploration of prospective biomarkers, with the aim of optimising personalised therapeutics against breast cancer.

## Methods

### Patients

This biomarker study used archived tumour specimens from patients with early-stage breast cancer who were enrolled in a prospective, multi-centre, single-arm neoadjuvant pyrotinib combined with trastuzumab treatment clinical trial, including its single-centre pilot trial (Chinese Clinical Trial Registry, ChiCTR1900022293. Registered on April 3, 2019). Patient eligibility was determined as previously described [17]. A total of 54 patients diagnosed between February 2019 to March 2021 at Southwest Hospital of Army Medical University were enrolled, including 14 samples from the pilot trial and 40 samples from the main trial. Four samples failed the quality control, resulting in a final total of 50 qualified sequencing analysis reports. Study protocols were approved by the Ethical Review Community of Southwest Hospital, and all patients signed informed consent forms in accordance with institutional guidelines.

### Treatment

Patients received four cycles of epirubicin (E) (100 mg/m^2^) and cyclophosphamide (C) (600 mg/m^2^) intravenously once every 3 weeks, followed by four cycles of docetaxel (T) (100 mg/m^2^) and trastuzumab (H) (8 mg/kg first load followed by 6 mg/kg) intravenously, once every 3 weeks. Pyrotinib (P) was administered orally at a dosage of 400 mg per day throughout the eight treatment cycles. Surgery was performed after the whole neoadjuvant course, and following surgery, the patients who reached a pCR continued to complete one year of trastuzumab treatment. For the patients who did not reach a pCR, the treatment strategy was altered in accordance with the clinician’s recommendation. The primary endpoint was total pCR, which was defined as the absence of invasive residual carcinoma in the breast and ipsilateral axillary lymph nodes (ypT0/is ypN0).

### DNA extraction, sequencing and analysis

Briefly, DNA extraction, library construction and sequencing, as well as a bioinformatics analysis, were performed. Firstly, the tissue DNA from formalin-fixed, paraffin-embedded (FFPE) tumour samples was extracted using a QIAamp DNA FFPE Tissue Kit (Qiagen, Hilden, Germany). Genomic DNA from the white blood cell samples was extracted using a DNeasy Blood & Tissue Kit (Qiagen) as the normal control of germline mutations. DNA was quantified by Qubit 3.0, and qualified samples proceeded to the NGS library construction step. A 250 ng sample of tissue DNA was sheared using Covaris M220 (Covaris, MA, USA), followed by end repair, A-tailing, and adaptor ligation of fragmented DNA. The fragments were purified by Agencourt AMPure XP beads (Beckman Coulter, CA, USA). Targeted enrichment was performed using a 425 cancer-related gene panel (Geneseeq Technology, Nanjing, China). The libraries captured by Dynabeads M-270 (Life Technologies, CA, USA) were amplified with KAPA HiFi HotStart ReadyMix (KAPA Biosystems, MA, USA). The purified library was quantified by quantitative polymerase chain reaction (qPCR) using a KAPA Library Quantification kit (KAPA Biosystems). Target enriched libraries were sequenced on Nextseq500 (Illumina, CA, USA) with 2 × 150 bp paired-end reads. Finally, bioinformatics analysis was performed, and the original sequencing data were demultiplexed by bcl2fastq (v2.19) and analysed by Trimmomatic [27] to remove low-quality data or N bases. Next, the data were aligned to the hg19 reference human genome using the Burrows-Wheeler Aligner algorithm [28]. The duplications were removed using the Picard suite [29]. Comprehensive genetic mutation information, including single nucleotide polymorphism (SNPs), insertions/deletions (indels), and copy number alterations (CNAs), were processed using a Genome Analysis Toolkit (GATK) [30].

### Tumour-infiltrating lymphocytes (TILs) evaluation

TILs of the archived biopsy samples before treatment of all patients were evaluated in accordance with the recommendations of the international TILs working group in 2014 [31], categorised as low (0–9%), intermediate (10–49%), and high (≥50%), respectively.

### Statistical analysis

The tumour mutation burden (TMB) between groups was compared with a Mann–Whitney *U* test. A comparison of the treatment response between groups was performed using a Fisher’s exact test, and only those genes with mutations or CNAs that exist in at least two patients were included in the comparations. Odds ratios and corresponding 95% CIs were calculated for all comparations. The Benjamini–Hochberg procedure was used to control the false discovery rate (FDR) in a multiple comparation procedure [32]. A Breslow–Day interaction test was applied to evaluate the homogeneity of the odds ratio between subgroups. A multivariate logistic regression analysis was constructed to combine the selected predictors for pCR, receiver operating characteristic (ROC), and corresponding area under curve (AUC) was applied to describe the discriminative performance of multivariate models. All analyses were performed using R 4.0.2 (http://www.r-project.org).

## Results

### Clinicopathological and TMB characteristics

**Fig. 1:**
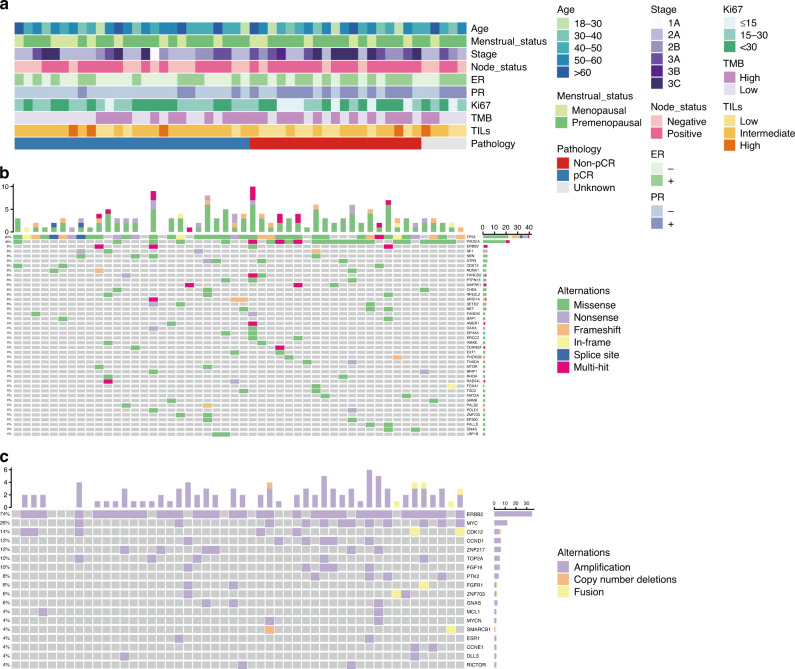
The clinical and genomic landscape of enrolled patients. **a** Clinicopathological characteristics; **b** somatic mutation profiling and **c** copy number alteration profiling. The figure only shows genes with mutations or copy number alterations that exist in at least two patients.

The baseline characteristics of all 50 patients are summarised in Supplementary Table S1 and visualised in Fig. 1a. The mean age of all enrolled patients was 48 years old, the relatively young age of the included patients was caused by both inclusion criterion of this prospective study (<70 years old) and Chinese women’s earlier age of breast cancer onset (with a peak at approximately 50 years). Out of these patients, five dropped out during treatment due to various reasons, whereas the other 45 patients completed the whole neoadjuvant chemotherapy course, as well as final surgery, among which 26 (52%) cases achieved total pCR (Supplementary Fig. S1). The median TMB was 4.76 mutations/Mb, and no significant difference was identified between the pCR and non-pCR populations in TMB (4.04 vs. 5.29 mutations/Mb). No microsatellite instability (MSI) was observed in the enrolled patients. As shown in Table 1, among all clinical features, a higher clinical stage indicates a worse response (46.7% vs. 80.0%, *P* = 0.039), patients with a positive lymph node (LN) status, positive hormone receptor (HR), and IHC 2+/FISH+ HER2 status exhibited a trend toward a worse treatment response but was not statistically significant (46.7% vs. 80.0%, *P* = 0.054; 43.5% vs. 72.7%, *P* = 0.071; 16.7% vs. 64.1%, *P* = 0.069, respectively), consistent with the findings of previous clinical researches [26, 33, 34]. Furthermore, higher levels of TILs in biopsy samples before treatment were more likely to achieve a pCR (*P* = 0.078), but not statistically significant.

**Table 1:** Univariate analysis of clinical features and response to pyrotinib combined with trastuzumab (HP) treatment.

Marker	pCR no. (%)	Non-pCR no. (%)	OR^a^ (95% CI)	*P*^b^
Age				0.485
<40	5 (45.5)	6 (54.5)		
≥40	21 (61.8)	13 (38.2)	1.91 (0.39−9.72)	
Menstrual status				1.000
Premenopausal	17 (56.7)	13 (43.3)		
Menopausal	9 (60.0)	6 (40.0)	1.14 (0.28−4.99)	
Lymph node				0.054
Positive	14 (46.7)	16 (53.3)		
Negative	12 (80.0)	3 (20.0)	4.42 (0.93−29.4)	
Clinical stage				0.039
I–II	18 (72.0)	7 (28.0)		
III	8 (40.0)	12 (60.0)	0.27 (0.06−1.06)	
Hormone receptor				0.071
Positive	10 (43.5)	13 (56.5)		
Negative	16 (72.7)	6 (27.3)	3.37 (0.85−14.75)	
HER2				
2+, FISH+	1 (16.7)	5 (83.3)		0.069
3+	25 (64.1)	14 (35.9)	8.51 (0.83−437.6)	
Ki67				1.000
≤30	15 (57.7)	11 (42.3)		
>30	11 (57.9)	8 (42.1)	1.01 (0.26−3.97)	
TILs				0.078
Low	4 (30.8)	9 (69.2)		
Intermediate	19 (67.9)	9 (32.1)	–	
High	3 (75.0)	1 (25.0)	–	

*pCR* pathologic complete response, *OR* odds ratio, *CI* confidence interval, *HER2* human epidermal growth factor receptor 2, *FISH* fluorescence in situ hybridisation, *TILs* tumour-infiltrating lymphocytes.

^a^The conditional maximum likelihood estimate (MLE) of the odds ratio was used.

^b^*P* value calculated using a Fisher’s exact test.

### Profiling of somatic genomic alterations

Among all of the enrolled patients, mutations were observed in 142 genes, of which the most frequently mutated driver genes consisted of TP53 (80%), PIK3CA (46%), ERBB2 (8%), NF1 (8%), NBN (8%), and ATRX (8%) (Fig. 1b). In terms of somatic CNAs, 43 different genes were identified with amplification, fusion, or copy number deletions. The most frequent amplification was ERBB2 (74%), followed by MYC (26%), CDK12 (14%), CCND1 (12%) and ZNF217 (12%) amplification (Fig. 1c). Gene fusion and copy number deletions were observed in 12 and 4 patients, respectively. It is worth noting that only 74% of cases have ERBB2 amplification detected by NGS, which indicates that HER2 overexpression defined by IHC or FISH is not fully equal to ERBB2 amplification [35].

### Association of PIK3CA mutations and pCR

**Fig. 2:**
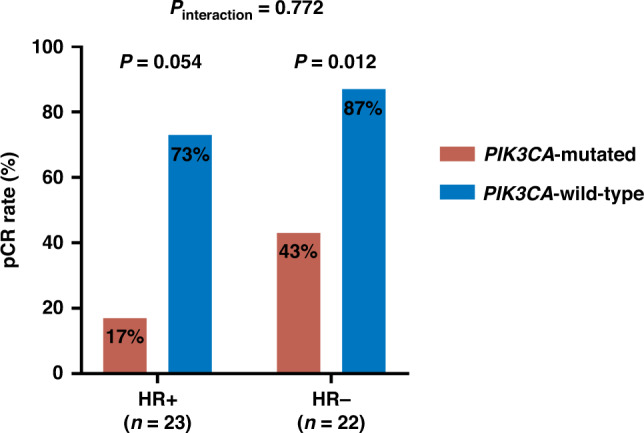
Subgroup analysis of pathological complete response (pCR). The pCR rates of PIK3CA-mutated and PIK3CA-wild-type patients stratified by the hormone receptor (HR) status.

Among all driver genes carried with mutations identified by NGS, only a PIK3CA mutation was screened out as having a significant relationship with the treatment response (Table 2). The pCR rate of patients with wild-type PIK3CA was significantly higher than in patients with mutated PIK3CA (80.8% vs. 26.3%; odds ratio (OR), 0.09; 95% CI: 0.02−0.35; *P* = 0.00057), and remained significant after a multiple comparation adjustment (*P*_adjusted_ = 0.024). Among the whole cohort, pCR was singularly achieved in five patients with a PIK3CA mutation. In contrast, another five patients with wild-type PIK3CA did not achieve a pCR and whose clinical features, including the postoperative Miller–Payne grade, are listed in Supplementary Table S2 with no identified special characteristics. Patients with PIK3CA mutations had a lower pCR rate independent of HR status (*P*_interaction_ = 0.772; Fig. 2; Supplementary Table S3). The specific mutation pattern of PIK3CA is presented in Supplementary Table S4, showing that 23 (46%) of the samples harboured activating mutations, among which three patients exhibited multi-hit mutations in PIK3CA. In addition, ERBB2 mutations were observed in four hotspots: I767M, L755S, E1168K and S609C. There were two patients who carried the I767M and L755S mutations are defined as a suspicious deleterious site that did not reach a pCR, whereas another two mutations remain functionally unclear to date [36].

**Table 2:** Univariate analysis of genetic alterations and response to pyrotinib combined with trastuzumab (HP) treatment.

Marker	pCR no. (%)	Non-pCR no. (%)	OR^a^ (95% CI)	*P*^b^	*P*_adjusted_^c^
PIK3CA				0.00057	0.024
Wild type	21 (80.8)	5 (19.2)			
Mutant	5 (26.3)	14 (73.7)	0.09 (0.02–0.42)		
TP53				0.704	1.000
Wild type	4 (50.0)	4 (50.0)			
Mutant	22 (59.5)	15 (40.5)	1.45 (0.23–9.15)		
ERBB2				1.000	1.000
Wild type	24 (58.5)	17 (41.5)			
Mutant	2 (50.0)	2 (50.0)	0.71 (0.05–10.75)		
NF1				0.627	1.000
Wild type	23 (56.1)	18 (43.9)			
Mutant	3 (75.0)	1 (25.0)	2.31 (0.17–129.9)		
NBN				0.627	1.000
Wild type	23 (56.1)	18 (43.9)			
Mutant	3 (75.0)	1 (25.0)	2.31 (0.17–129.9)		
ATRX				1.000	1.000
Wild type	24 (58.5)	17 (41.5)			
Mutant	2 (50.0)	2 (50.0)	0.71 (0.05–10.75)		
MYC				0.010	0.183
Neutral	24 (68.6)	11 (31.4)			
Amplification	2 (20.0)	8 (80.0)	0.12 (0.01–0.74)		
ERBB2				0.734	1.000
Neutral	6 (50.0)	6 (50.0)			
Amplification	20 (60.6)	13 (39.4)	1.52 (0.33–7.14)		
CDK12				0.686	1.000
Neutral	23 (59.0)	16 (41.0)			
Amplification	3 (50.0)	3 (50.0)	0.70 (0.08–5.92)		
CCND1				0.069	0.412
Neutral	1 (16.7)	5 (83.3)			
Amplification	25 (64.1)	14 (35.9)	0.12 (0.002–1.20)		
ZNF217				1.000	1.000
Neutral	22 (56.4)	17 (43.6)			
Amplification	4 (66.7)	2 (33.3)	1.53 (0.19–18.81)		

*pCR* pathologic complete response, *OR* odds ratio, *CI* confidence interval.

^a^The conditional maximum likelihood estimate (MLE) of odds ratio was used.

^b^*P* value was calculated by Fisher’s exact test.

^c^Adjusted *P* value for multiple comparisons by the Benjamini–Hochberg method.

### Association of MYC copy number alterations and pCR

Among all genes with CNAs identified in these patients, only MYC amplification was found to exhibit a significant relationship with lower pCR rate (68.6% vs. 20.0%; OR, 0.12; 95% CI: 0.01−0.74; *P* = 0.010), while this distinction turned out to be non-significant after correction for multiple comparation (*P*_adjusted_ = 0.183) (Table 2). It has generally been established that amplification of ERBB2 leads to the overexpression of its receptor; however, as a potential predictor, ERBB2 amplification was found to have no association with pCR (*P* = 0.734).

### Combination of genetic and clinical predictors of pCR

**Fig. 3:**
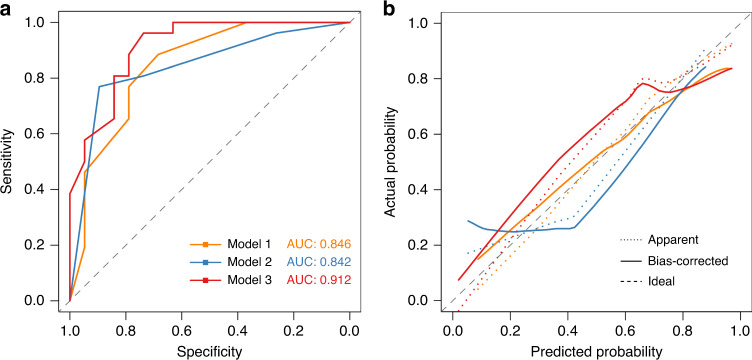
Performance evaluation of three multivariate logistic regression models. **a** The receiver operator characteristic (ROC) curve describing discrimination of the models. Sensitivity-(1 − Specificity) curves were plotted by different cut-off points; a higher area under curve indicates better discrimination. **b** Calibration curves that evaluate the consistency of models. Model-predicted pCR probability is plotted on the X-axis and the actual probability is plotted on the *Y* axis. The dotted grey line (plotted by function *y* = *x*) indicates the perfect match of actual and predicted results.

The predictive value with a multivariate logistic regression model of either clinical features, genetic biomarkers, or both were evaluated (Table 3). Model 1 combined the four clinical features (HR status, LN status, clinical stage and TILs) with a *P* value less than 0.1 in the univariate analysis, and only HR status was a significant predictor for pCR (*P* = 0.046). Model 2 considered two genetic biomarkers, in which both PIK3CA mutations (*P* = 0.0015) and MYC amplification (*P* = 0.024) were significant predictors. In model 3, both clinical features and genetic biomarkers were integrated, and only the presence of a PIK3CA mutation was a strong predictor for a pCR (*P* = 0.0069). The ROC curves (Fig. 3a) showed that among the three models, model 3 had the greatest predictive performance with a relatively higher AUC of 0.912 (95% CI: 0.827−0.997). The calibration plots verified good consistency between the actual and model-predicted pCR probability, especially for model 3 (Fig. 3b). A nomogram predicting pCR probability based on model 3 was also established (Supplementary Fig. S2).

**Table 3:** Multivariate logistic regression model of the treatment response for selected variables.

Variable	OR (95% CI)	*P*	AUC (95% CI)
Model 1: Prediction with clinical features			0.846 (0.724–0.968)
HR status (negative vs. positive)	5.52 (1.15–36.40)	0.046	
LN status (negative vs. positive)	4.09 (0.65–36.40)	0.148	
Clinical stage (III vs. I–II)	0.28 (0.04–1.53)	0.156	
HER2 (3+ vs. 2+/FISH+)	4.03 (0.31–120.5)	0.324	
TILs (intermediate/high vs. low)	5.10 (0.99–33.52)	0.063	
Model 2: Prediction with genetic biomarkers			0.842 (0.728–0.957)
PIK3CA (mutant vs. WT)	0.08 (0.01–0.34)	0.0015	
MYC (amplification vs. neutral)	0.10 (0.01–0.63)	0.024	
Model 3: Prediction with combined predictors			0.912 (0.827–0.997)
HR status (negative vs. positive)	2.65 (0.32–26.65)	0.367	
LN status (negative vs. positive)	4.42 (0.49–59.21)	0.212	
Clinical stage (III vs. I–II)	0.45 (0.04–4.68)	0.502	
TILs (intermediate/high vs. low)	6.33 (0.71–91.11)	0.120	
HER2 (3+ vs. 2+/FISH+)	10.54 (0.41–648.7)	0.200	
PIK3CA (mutant vs. WT)	0.07 (0.01–0.41)	0.007	
MYC (amplification vs. neutral)	0.22 (0.02–2.27)	0.211	

*OR* odds ratio, *CI* confidence interval, *TILs* tumour-infiltrating lymphocytes, *WT* wild-type, *AUC* area under curve, *HR* hormone receptor, *LN* lymph node.

## Discussion

In this biomarker analysis, targeted NGS across 425 cancer-related genes was used to identify candidate genetic biomarkers for predicting the response to neoadjuvant pyrotinib plus trastuzumab dual anti-HER2 treatment. First, we revealed that PIK3CA mutations were associated with a reduced treatment response to pyrotinib-containing dual anti-HER2 neoadjuvant in 50 participants. Moreover, there were no positive findings with regards to the other genetic biomarkers except for PIK3CA mutations, including TMB and ERBB2 amplification. MYC amplification was found to be associated with a lower pCR rate in unadjusted comparations; however, the possibility that the difference was derived from an increased probability of a type I error occurring in multiple comparations cannot be excluded. In addition, the relationship between MYC amplification and the treatment response still requires verification. Based on a multivariate logistic regression model that integrated PIK3CA, MYC, and selected clinical features, improved performance was achieved for predicting a pCR in patients in this therapeutic environment, with an AUC of 0.896 (95% CI: 0.795−0.997). This may aid in predicting the patient response before selecting a patient for this treatment.

It has been generally established that the p110α catalytic subunit of PI3K is encoded by the PIK3CA gene, which phosphorylates phosphatidylinositol-4,5-bisphosphate (PIP2) at the third position of the inositol ring, generating PIP3, after recruitment to the cellular membrane via receptor-mediated activation. Subsequently, Akt is phosphorylated, stimulating a signalling cascade, including mammalian target of rapamycin (mTOR) and downstream effectors [37]. Hotspot mutations in the helical or kinase domains hyperactivate the PI3K/Akt pathway downstream of the ErbB family [38], among which H1047R, E545K, and E542K are the most common mutation sites, accounting for ~63% of all PIK3CA mutations [39]. Two rigorous research letters reported that H1047R mutation in PIK3CA induces multipotency and multi-lineage mammary tumours [40], and induced multipotency in unipotent progenitors [41]. It was further found that double PIK3CA mutations in *cis* activate PI3K pathway cellular signalling and promote growth more than single mutants [42]. These interesting findings in experimental research need to be linked to clinical practice. A recent study involving a cohort containing 412 Chinese patients with invasive breast cancer revealed that Chinese patients had a higher PIK3CA alteration frequency (45.6% vs. 34.7%; *P* < 0.001) compared to Caucasians in The Cancer Genome Atlas (TCGA) (*N* = 453), indicating that an alteration in PIK3CA deserves greater attention among Chinese patients [43]. PIK3CA mutations have also been observed more frequently in the luminal type than in the basal-like breast cancers. In the above cohort, PIK3CA alterations occurred at the highest frequency in ER+/HER2+ (51.6%) tumours across four subtypes. The in vitro data suggested that aberrant activation of the PI3K/Akt pathway via activating PIK3CA mutations was implicated in resistance to targeted anti-HER2 trastuzumab [44, 45]; however, the mechanisms associated with how PIK3CA mutations modulating the efficacy of ErbB-targeted TKI are less well-established.

Several clinical trials have demonstrated that PIK3CA mutations are associated with a pCR rate arising from dual anti-HER2 neoadjuvant therapy. In a Phase III NeoALTTO trial, 114 patients with early HER2-positive breast cancer were enrolled and administered a combination of trastuzumab and lapatinib. Eventually, a pCR rate of 53.1% was obtained in patients who had wild-type PIK3CA, whereas the rate was attenuated to 28.6% in those who carried PIK3CA mutations (*P* = 0.012) [33]. Similarly, PIK3CA mutations were significantly associated with a lower pCR rate in the patients who received dual anti-HER2 treatment in the GeparSixto trial (17.4% vs. 37.1%; *P* = 0.013) and CHER-LOB trial (12.5% vs. 48.4%; *P* = 0.06) [26, 46]. A pooled analysis that combined individual patient data from five clinical trials evaluating neoadjuvant therapy with trastuzumab and/or lapatinib in addition to taxane-based chemotherapy also concluded that an overall pCR rate was significantly lower in the patient population with PIK3CA mutations [25].

We summarised studies on response to TKI treatment of HER2-positive breast cancers according to PIK3CA mutation status (Supplementary Table S5), almost all the previous studies explored the association of PIK3CA mutations and resistance to lapatinib, while evidence for pyrotinib, a new pan-ErbB TKI with the capacity to irreversibly block ErbB1, ErbB2 and ErbB4, was absent. Pyrotinib and lapatinib are different in mechanism, including binding sites and reversibility; hence, they are different in clinical efficacy. For example, in a recent Phase III randomised controlled trial (PHOEBE), pyrotinib plus capecitabine led to a statistically and clinically significant improvement in progression-free survival in HER2-positive metastatic breast cancers compared with lapatinib plus capecitabin [13]. Therefore, whether there is a difference between pyrotinib and lapatinib in resistance status in HER2 targeted therapy was unknown, and it is hard to directly apply the conclusions of previous studies or meta-analyses to the new therapeutic environment.

The potential relationship between PIK3CA and the response to pyrotinib treatment was firstly indicated in a metastatic setting in 2017, which reported that PIK3CA and TP53 double mutations in circulating tumour DNA (ctDNA) is a potential predictor for the insensitive treatment response of pyrotinib (*P* = 0.013); however, this conclusion requires further evaluation given the limited sample size (*n* = 18), and lack of data concerning PIK3CA mutation alone [47]. In another retrospective real-world study, performing an NGS analysis on the ctDNA of 28 metastatic patients who were administered treatment with pyrotinib combined with other drugs, and failed to identify a correlation between the PIK3CA status and PFS (*P* = 0.515) [48]. The sample size of 50 cases may be a limitation of our study. Nevertheless, the conclusion is statistically robust because the significance was identified after a multiple comparison adjustment. The result is meaningful as suggestive evidence in clinical practice for patient preselection before using pyrotinib and trastuzumab dual HER2-targeted therapy. However, it is necessary to improve the reliability of our conclusion by expanding the sample size or combining our data with other similar data in meta-analysis in future works.

Furthermore, in terms of HR status subgroups, patients who harboured HR+ and PIK3CA-mutant cancer appeared to have a significantly lower pCR rate and worse DFS [26, 33]. In this study, although no interaction between HR status and PIK3CA mutation was identified, HR+/HER2+ tumours harbouring PIK3CA mutations numerically had the lowest pCR rate (17%, 2 of 12), which is consistent with the findings of the above studies. In terms of specific mutation patterns, these three hotspot mutations accounted for 66% of all PIK3CA mutations: H1047R (44%), E545K (15%), E542K (7%) in our research. Of note, the pCR rate of patients harbouring H1047R mutation in PIK3CA is 20% (2/10), and the four available patients harbouring E545K or E542K mutation did not achieve pCR. The three patients harbouring double or treble PIK3CA mutations also all failed to achieve pCR. Of course, given our small sample size, the effect of specific PIK3CA mutations needs clinical validation. However, these data are suggestive for clinical treatment and future studies, for example, the use of PI3K inhibitors in HER2+, PIK3CA-mutant breast cancers.

At present, the first PI3K p110α inhibitor, alpelisib, has acquired FDA approval in 2019 in patients with HR-positive, HER2-negative, PIK3CA-mutant metastatic breast cancer based on the SOLAR-1 trial [49]. Several preclinical and clinical studies investigating new strategies based on PI3K inhibitors combined with anti-HER2 therapy in HER2-positive and PIK3CA-mutant patients are underway and will explain to what extent can PI3K inhibitors improve the treatment response of PIK3CA-mutant tumours that are resistant to anti-HER2 therapy [50].

Despite its novelty, our study still has some limitations that should be noted. Firstly, the average age of our cohort is relatively low (48 years old), because the selection criteria of ChiCTR1900022293 allow the inclusion up to 70 years old patient, which may affect the external validity of our findings. Secondly, the discordance exists in HER2 overexpression determined by IHC or FISH and ERBB2 amplification detected by NGS, only 74% of HER2-overexpression cases are determined as ERBB2 amplification. Even though we have proved that ERBB2 amplification status do not have effect on treatment response in HER2-expression cohort according to univariate analysis, it should be especially noticed that ERBB2 amplification is not equivalent to HER2 overexpression in patient selection. Thirdly, based on the exploration nature of our clinical study, it’s hard to make external validation in our multivariate model because the drug is only approved in metastatic setting at current stage.

In conclusion, the present study demonstrated that HER2-positive breast cancers with activating mutations in PIK3CA are less likely to benefit from pyrotinib combined with trastuzumab neoadjuvant therapy. Our data support the clinical significance of ongoing studies of the combination of anti-HER2 therapy and PI3K inhibitors.

## Supplementary information

Figure S1

Figure S2

Table S1

Table S2

Table S3

Table S4

Table S5

aj-checklist

## Data Availability

All the data generated and/or analysed during this study are available from the corresponding author upon reasonable request.

## Code availability

All the code programmed during this study are available from the corresponding author upon reasonable request.
