# Supplementary figures and images for: PIK3CA mutations are associated with pathologic complete response rate to neoadjuvant pyrotinib and trastuzumab plus chemotherapy for HER2-positive breast cancer
Source: Br J Cancer. 2022 Nov 2;128(1):121–9. doi: 10.1038/s41416-022-02021-z (PMC9814131; doi:10.1038/s41416-022-02021-z)

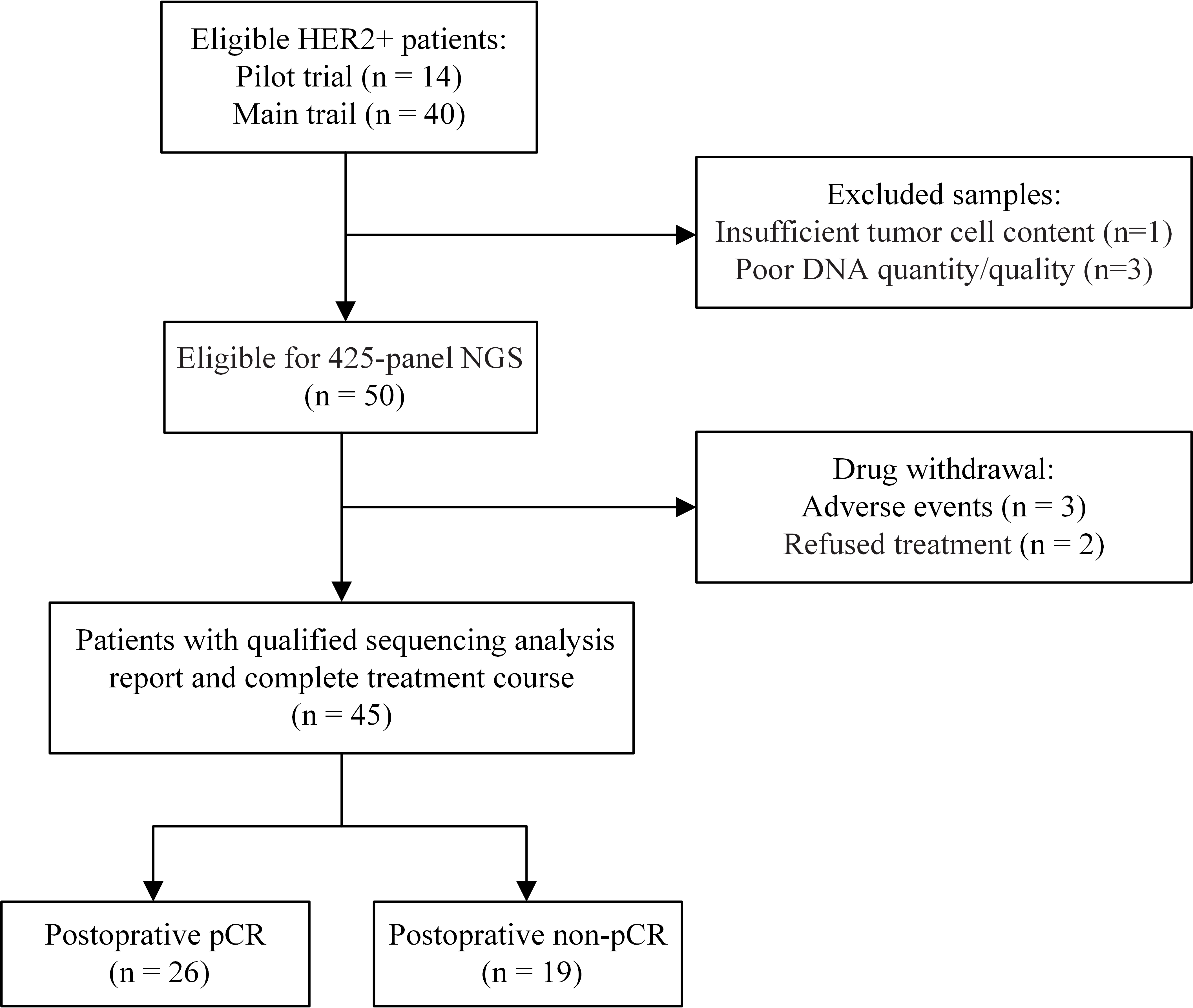


**Figure S1**. Diagram of sample selection used for biomarker analysis.

Supplement: Supplementary file 1 — Figure S1 [file 41416_2022_2021_MOESM1_ESM.docx]
