# Supplementary material for: PIK3CA mutations are associated with pathologic complete response rate to neoadjuvant pyrotinib and trastuzumab plus chemotherapy for HER2-positive breast cancer
Source: Br J Cancer. 2022 Nov 2;128(1):121–9. doi: 10.1038/s41416-022-02021-z (PMC9814131; doi:10.1038/s41416-022-02021-z)
Supplement: Supplementary file 2 — Figure S2 [file 41416_2022_2021_MOESM2_ESM.docx]

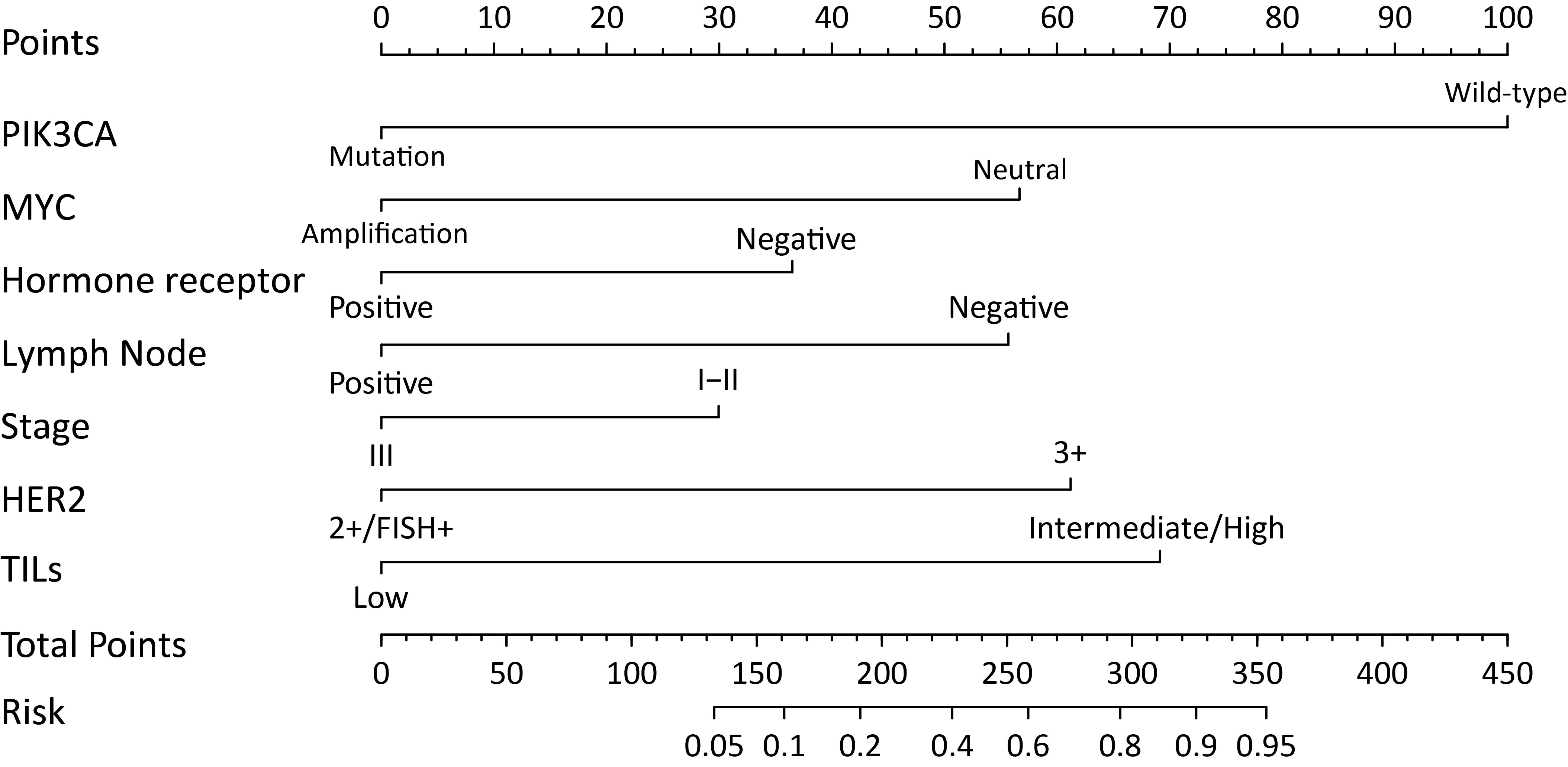


**Figure S2.** Nomogram of Model 3 for predicting the pathological complete response (pCR). Each variable corresponds to a score on the points scale. After adding up the total points, the predicted pCR possibility could be obtained by projecting the total points to the risk axis.
