# Supplementary material for: PIK3CA mutations are associated with pathologic complete response rate to neoadjuvant pyrotinib and trastuzumab plus chemotherapy for HER2-positive breast cancer
Source: Br J Cancer. 2022 Nov 2;128(1):121–9. doi: 10.1038/s41416-022-02021-z (PMC9814131; doi:10.1038/s41416-022-02021-z)
Supplement: Supplementary file 3 — Table S1 [file 41416_2022_2021_MOESM3_ESM.docx]

**Table S1.** Clinicopathological and tumor mutation burden (TMB) characteristics.

| **Characteristics** | **N = 50** |
| --- | --- |
| Age, mean (SD), years | 48.00 (10.88) |
| Menstrual status, n (%) |  |
| Premenopausal | 34 (68) |
| Menopausal | 16 (32) |
| Lymph node status, n (%) |  |
| Positive | 32 (64) |
| Negative | 18 (36) |
| Clinical stage, n (%) |  |
| I | 1 (2) |
| II | 28 (56) |
| III | 21 (42) |
| ER, n (%) |  |
| Positive | 25 (50) |
| Negative | 25 (50) |
| PR, n (%) |  |
| Positive | 19 (38) |
| Negative | 31 (62) |
| HER2, n (%) |  |
| 2+, FISH + | 7 (14) |
| 3+ | 43 (86) |
| Ki-67, n (%) |  |
| <=30 | 29 (58) |
| >30 | 21 (42) |
| TILs, n (%) |  |
| Low | 15 (30) |
| Intermediate | 30 (60) |
| High | 5 (10) |
| TMB, range, mutations/Mb | 4.76 (0.00, 29.61) |
| Postoperative pathology, n (%) |  |
| pCR | 26 (52) |
| Non-pCR | 19 (38) |
| Unfinished^a^ | 5 (10) |

Abbreviations: SD, standard deviation; ER, estrogen receptor; PR, progesterone receptor; HER2, human epidermal growth factor receptor 2; FISH, fluorescence in situ hybridization; TILs, Tumor-infiltrating lymphocytes; TMB, tumor mutation burden; pCR, pathologic complete response.

^a^ Patients who dropped out during treatment due to any reason and did not finish surgery.
