# Supplementary material for: PIK3CA mutations are associated with pathologic complete response rate to neoadjuvant pyrotinib and trastuzumab plus chemotherapy for HER2-positive breast cancer
Source: Br J Cancer. 2022 Nov 2;128(1):121–9. doi: 10.1038/s41416-022-02021-z (PMC9814131; doi:10.1038/s41416-022-02021-z)
Supplement: Supplementary file 4 — Table S2 [file 41416_2022_2021_MOESM4_ESM.docx]

**Table S2:** Clinicopathologic details of pCR patients with mutated PIK3CA (n = 5) and non-pCR patients with wild-type PIK3CA (n = 5).

| **Patient ID** | **Age** | **Menstrual status** | **Lymph node** | **Clinical stage** | **Hormone receptor** | **HER2** | **Ki67** | **Miller-Payne grade** |
| --- | --- | --- | --- | --- | --- | --- | --- | --- |
| pCR patients with mutated PIK3CA (n = 5) | | | | | | | | |
| 001 | 65 | Menopausal | - | 2A | + | 3+ | 30 | 5 |
| 020 | 69 | Premenopausal | + | 3C | - | 3+ | 30 | 5 |
| 029 | 43 | Premenopausal | - | 1A | + | 2+/FISH+ | 50 | 5 |
| 032 | 63 | Menopausal | - | 2A | + | 3+ | 20 | 5 |
| 048 | 49 | Premenopausal | + | 3A | - | 3+ | 40 | 5 |
| Non-pCR patients with wild-type PIK3CA (n = 5) | | | | | | | | |
| 011 | 56 | Menopausal | - | 2B | + | 3+ | 60 | 4 |
| 016 | 60 | Menopausal | + | 3A | + | 2+/FISH+ | 30 | 3 |
| 038 | 36 | Premenopausal | - | 3A | - | 3+ | 60 | 3 |
| 039 | 41 | Premenopausal | + | 2B | + | 2+/FISH+ | 70 | 2 |
| 043 | 46 | Premenopausal | + | 3C | - | 2+/FISH+ | 30 | 2 |

Abbreviations: pCR, pathologic complete response; HER2, human epidermal growth factor receptor 2; FISH, fluorescence in situ hybridization.
