# Supplementary material for: PIK3CA mutations are associated with pathologic complete response rate to neoadjuvant pyrotinib and trastuzumab plus chemotherapy for HER2-positive breast cancer
Source: Br J Cancer. 2022 Nov 2;128(1):121–9. doi: 10.1038/s41416-022-02021-z (PMC9814131; doi:10.1038/s41416-022-02021-z)
Supplement: Supplementary file 5 — Table S3 [file 41416_2022_2021_MOESM5_ESM.docx]

**Table S3.** PIK3CA mutation status and pCR by hormone receptor status.

| **PIK3CA Status** | **pCR No. (%)** | **Non-pCR No. (%)** | ***P*^a^** |
| --- | --- | --- | --- |
| HR-negative |  |  | 0.054 |
| Wild-type | 13 (87) | 2 (13) |  |
| Mutant | 3 (43) | 4 (57) |  |
| HR-positive |  |  | 0.012 |
| Wild-type | 8 (73) | 3 (27) |  |
| Mutant | 2 (17) | 10 (83) |  |

Abbreviations: pCR, pathologic complete response; HR, hormone receptor.

^a^ *P* value was calculated using a Fisher’s exact test.
