# Supplementary material for: PIK3CA mutations are associated with pathologic complete response rate to neoadjuvant pyrotinib and trastuzumab plus chemotherapy for HER2-positive breast cancer
Source: Br J Cancer. 2022 Nov 2;128(1):121–9. doi: 10.1038/s41416-022-02021-z (PMC9814131; doi:10.1038/s41416-022-02021-z)
Supplement: Supplementary file 6 — Table S4 [file 41416_2022_2021_MOESM6_ESM.docx]

**Table S4.** PIK3CA Mutation Frequency.

| **Exon** | **Amino acid** | **Nucleotide** | **p110α domain** | **n** | **%** |
| --- | --- | --- | --- | --- | --- |
| 20 | p.H1047R^a^ | c.3140A>G | Catalytic | 12 | 44.4 |
| 9 | p.E542K | c.1624G>A | Helical | 4 | 14.8 |
| 9 | p.E545K | c.1633G>A | Helical | 2 | 7.4 |
| 1 | p.E81K^a^ | c.241G>A | ABD | 1 | 3.7 |
| 2 | p.D186H^a^ | c.556G>C | NA | 1 | 3.7 |
| 7 | p.E453Q^a^ | c.1357G>C | C2 | 1 | 3.7 |
| 9 | p.E522Q^a^ | c.1564G>C | NA | 1 | 3.7 |
| 9 | p.E545A | c.1634A>C | Helical | 1 | 3.7 |
| 9 | p.Q546P | c.1637A>C | Helical | 1 | 3.7 |
| 9 | p.Q546R | c.1637A>G | Helical | 1 | 3.7 |
| 20 | p.E982K | c.2944G>A | Catalytic | 1 | 3.7 |
| 20 | p.D1017H^a^ | c.3049G>C | Catalytic | 1 | 3.7 |

Abbreviations: ABD, adaptor-binding domain; NA, not available; C2, cellular membranes-binding domain.

* Multi-hit mutations co-exist with other locations
