# Supplementary material for: PIK3CA mutations are associated with pathologic complete response rate to neoadjuvant pyrotinib and trastuzumab plus chemotherapy for HER2-positive breast cancer
Source: Br J Cancer. 2022 Nov 2;128(1):121–9. doi: 10.1038/s41416-022-02021-z (PMC9814131; doi:10.1038/s41416-022-02021-z)
Supplement: Supplementary file 7 — Table S5 [file 41416_2022_2021_MOESM7_ESM.docx]

**Table S5:** Summary of the studies on response to TKI treatment of HER2-positive breast cancers according to PIK3CA mutation status.

| Study name/ID | Year | Country | Stage | Sample size (TKI-containing groups) | Original study design | Anti-HER2 regimen | Endpoints |
| --- | --- | --- | --- | --- | --- | --- | --- |
| NCT00206427 | 2011 | USA | IIIb-IV | 31 | Phase II, single-arm, open-label, L→T+H | Trastuzumab and Lapatinib | pCR |
| GeparQuinto  (NCT00567554) | 2014 | Germany | II-III | 91 | Phase III, randomized, open-label, AC+H→T+H vs. AC+L→T+L | Trastuzumab or Lapatinib | pCR |
| GeparSixto  (NCT01426880) | 2014 | Germany | II-III | 240 | Phase II, randomized, open-label, AT+HL vs. ATCb+HL | Trastuzumab and Lapatinib | pCR  DFS  OS |
| NeoALTTO  (NCT00553358) | 2015 | USA | II-III | 234 | Phase III, randomized, open-label, T+H vs. T+L vs. T+ HL | Trastuzumab and/or Lapatinib | pCR  EFS  OS |
| DAFNE(GBG-70)  (NCT01594177) | 2015 | Germany | II-III | 61 | Phase II, single-arm, open-label, HAf→T+HAf→AC+H | Trastuzumab and Afatinib | pCR |
| CHER-LOB  (NCT00429299) | 2015 | Italy | II-IIIa | 75 | Phase II, randomized, open-label, T+H→FAC+H vs. T+L→FAC+L vs. T+HL→FAC+HL | Trastuzumab and/or Lapatinib | pCR  RFS  OS |
| ICORG 10-05  (NCT01485926) | 2017 | Ireland | II-III | 39 | Phase II, randomized, open-label, TCb+H vs. TCb+L vs. TCb+HL | Trastuzumab and/or Lapatinib | pCR |
| TBCRC006  (NCT00548184) | 2018 | USA | III-III | 46 | Phase II, single-arm, open-label, HL+endocrine therapy (ER+) | Trastuzumab and Lapatinib | pCR |
| ExteNET  NCT00878709 | 2019 | Canada | II-III | 489 | Phase III, randomized, double-blind, N vs. placebo | Neratinib | iDFS |
| NCT00338247 | 2011 | China | IV | 57 | Phase II, single-arm, open-label, Cb+L, previously treated with A, T and H | Lapatinib | ORR  CBR  PFS  OS |
| EGF109491  (NCT00508274) | 2011 | China | IV | 38 | Phase III, single-arm, open-label, X+L, previously treated with A, T and H | Lapatinib | ORR  CBR  PFS  TTR  DoR |
| EGF104535  (NCT00281658) | 2014 | China | IV | 87 | Phase III, randomized, open-label, T+L vs. T, first-line | Trastuzumab ±Lapatinib | ORR  CBR  OS  PFS |
| EMILIA  (NCT00829166) | 2016 | Germany | IV | 98 | Phase III, randomized, open-label, T-DM1 vs. X+L, previously treated with T and H | Lapatinib | ORR |
| UMIN000007153 | 2017 | Japan | IV | 69 | Phase II, single-arm, open-label, X+L, previously treated with T and H | Lapatinib | ORR  PFS  OS |
| WJOG6110B/ELTOP  (UMIN000005219) | 2018 | Japan | IV | 16 | Phase II, randomized, open-label, X+H vs. X+L, previously treated with T and H | Trastuzumab or Lapatinib | ORR  PFS |
| NALA (NCT01808573) | 2021 | Spain | IV | 420 | Phase III, randomized, open-label, X+N vs. X+L, previously received two or more HER2-targeted therapies | Neratinib or Lapatinib | PFS |

Abbreviations: TKI, tyrosine kinase inhibitor; L, lapatinib; T, taxanes; H, trastuzumab; pCR, pathologic complete response; A, anthracyclines; C, cyclophosphamide; Cb, carboplatin; DFS, disease free survival; OS, overall survival; EFS, event free survival; Af, afatinib; F, Fluorouracil; RFS, relapse free survival; ER, estrogen receptor; N, neratinib; iDFS, invasive disease-free survival; ORR, objective response rate; CBR, clinical benefit rate; X, capecitabine; TTR, time to response; DoR, duration of response.
